# Domestication of wild animals may provide a springboard for rapid variation of coronavirus

**DOI:** 10.1515/biol-2021-0027

**Published:** 2021-03-16

**Authors:** Lei Gao

**Affiliations:** School of Life Sciences, South China Normal University, No. 55 Zhongshan Avenue West, Tianhe District, Guangzhou, 510631, Guangdong Province, China

**Keywords:** coronavirus, wild animal domestication, intermediate hosts, pangolin

## Abstract

Coronaviruses have spread widely among humans and other animals, but not all coronaviruses carried by specific animals can directly infect other kinds of animals. Viruses from most animal hosts need an intermediate host before they can spread widely among humans. Under natural conditions, coronaviruses do not rapidly change from infecting wild animals as intermediate hosts and to spreading widely among humans. The intermediate host might be the animals captured or bred for the purpose of cross-breeding with domesticated species for improvement of the breed. These animals differ from wild animals at the environmental and genetic levels. It is an important direction to study the semi-wild animals domesticated by humans in search for intermediate hosts of viruses widely spread among humans.

Coronaviruses are common in humans and other animals. Because of the large number of variants, they pose a great threat to human and animal health. Like all other viruses, they cannot live independently and require living bacteria or other cells to reproduce. When a virus infects a bacteria or other host cell, it must first solve the problem of recognition between the virus and the host [1]; only viruses that can bind to a host receptor can infect the host [2]. Studies have shown that hosts that can be parasitized or infected by coronaviruses are usually specific; that is, the coronavirus host usually has a typical receptor [3]. In general, the relationship between virus and host is often interdependent. In the long-term evolutionary process, the virus can come to coexist with the host indefinitely, because a virus that kills its host will die as well. Conversely, viruses with strong pathogenicity to a specific organism are usually new viruses. These types of viruses have not been tempered by co-evolution with the hosts over a long time.

In most cases, because of the specificity of the recognition mechanism between viruses and hosts, the viruses carried by a certain kind of animal host often cannot infect other kinds of animals. That is because the virus receptors in different groups of organisms are not the same [4]. For example, although the new coronavirus may have originated from a type of bat, the coronavirus carried by the bat cannot directly infect humans [5]. If a coronavirus can infect a new host different from the current host, it must undergo a large number of mutations [6]. Under natural conditions, this transmission of coronavirus is not common among distant species [7]. Although animal predations and human hunting behaviors have existed for a long time, it is not typical for a large-scale viral infection to occur in predators or hunters and spread widely. For a virus to be able to infect across species, it must mutate its host recognition mechanism and successfully avoid immunity from the new host. The virus must undergo rapid mutation to adapt to the new host. Therefore, before coronavirus can successfully infect humans, it must complete mutation with the help of intermediate hosts and gradually evolve into a new variant of the virus that can be identified by human receptors. Scientists have speculated that the intermediate host of new coronaviruses may be a kind of pangolin [8] because studies have shown that a variant of coronavirus carried by a certain type of bat (*Rhinolophus*) maybe the origin of the new coronavirus because of the similar gene sequences [9]. However, the relationship between pangolin and humans is not close in the phylogenetic linage. Bats and pangolins are wild animals, so they may have a relatively stable relationship in natural states (e.g., pangolins may feed on bat feces, or their path tracks may cross) [10]. Therefore, in the long process of animal and virus evolution, the new coronavirus carried by the bat should not be transmitted to pangolins until now. For this reason, if there is a new virus mutation, there must either be a new species, or the type of intermediate host has changed rapidly. When the host changes, the virus undergoes rapid selective evolution [11,12]. Therefore, when a new virus with strong infectivity or pathogenicity suddenly appears, its intermediate host might be a new variant of a certain kind that has changed the living environment conditions rather than a known wild species.

It is known that under natural conditions, the evolutionary variation of species is slow, and so most of the viruses do not undergo dramatic evolutionary selection. However, human participation in the artificial selection of wild species can significantly accelerate the rate of species variation and evolution. In particular, with animals that can be eaten for their meat, people generally use large-scale artificial breeding, including cross-breeding with related species of livestock, to improve the characteristics of domestic animals. The living environment and dietary structure of these animals are quite different from those of the previous wild environment. Even those improved varieties made by cross-breeding with domestic animals are significantly different from wild animals at the genetic level. To adapt to the new environment, the viruses carried by these animals will also undergo rapid mutation and evolution [10]. In the process of raising these animals, humans engage in widespread contact with or eat these animals, which increases the chance of the virus entering the human body. In this way, captive-bred wild animals and hybrid varieties through domesticated animals crossbred with wild animals may become an essential intermediate host for the virus carried by wild animals, and so these animals will become critical stepping stones for the virus to infect humans. Similarly, we cannot rule out that the intermediate host of the new coronavirus, which is now widely spread in the population, might be wild animals in artificial breeding or improved varieties hybridized with domestic animals. Therefore, it is difficult to understand the direct transmission of new coronavirus from bats or wild pangolins to humans (Figure 1).

**Figure 1:**
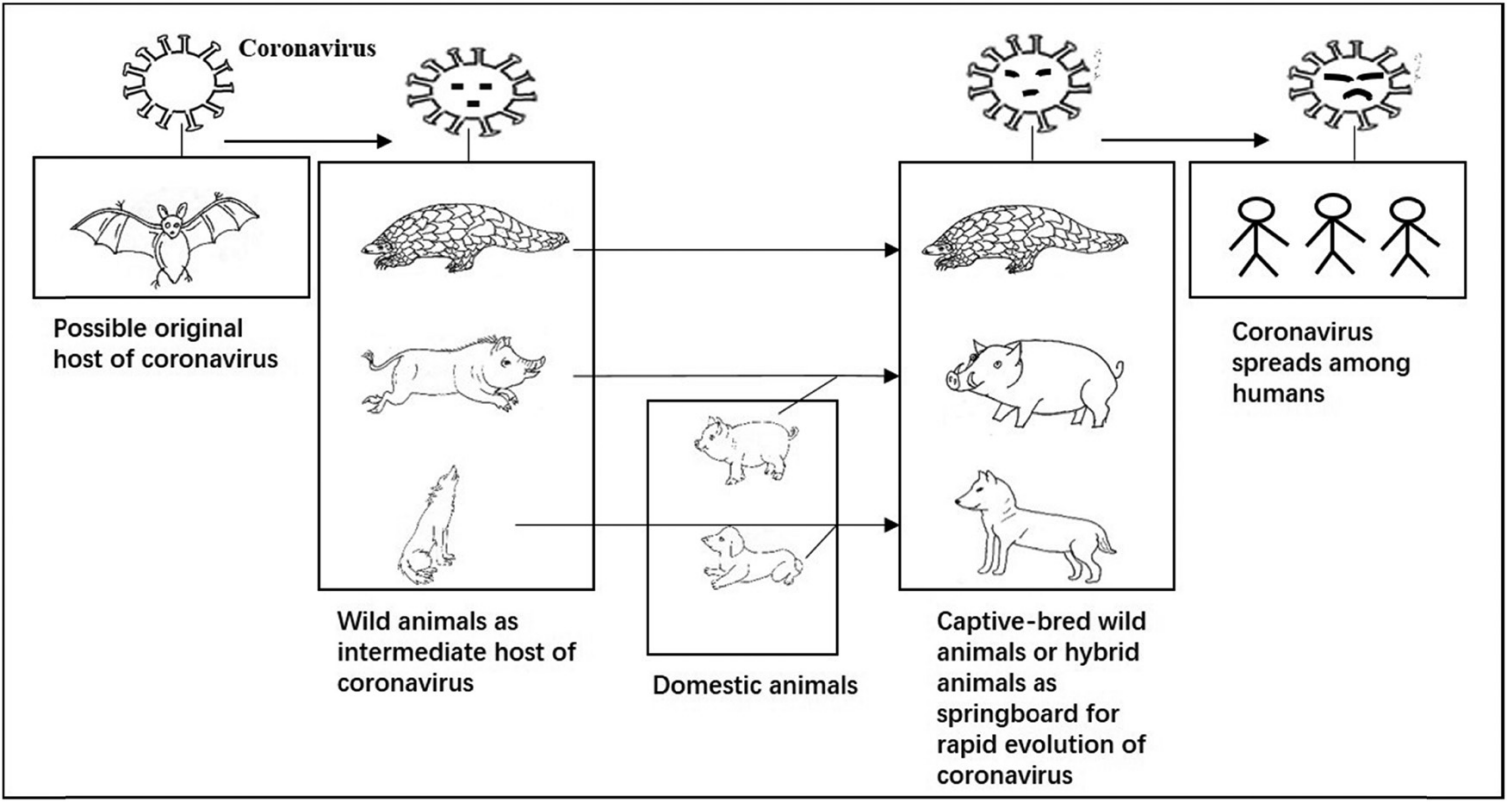
A possible transmission route of new coronavirus from the original host to humans. It was shown that captive-bred wild animals and hybrid varieties through domesticated animals crossbred with wild animals might be the intermediate host of coronavirus from wild animals to humans. In addition, it means that the coronavirus evolves rapidly with the changes of its hosts so that the coronavirus could eventually spread among humans, while the coronavirus carried by the original host cannot be directly transmitted to humans.

Although some viruses can spread directly from wild animals to humans or cause outbreaks of zoonotic infections, such as SARS, MERS, and Ebola, these viruses lead to episodic outbreaks. However, when we trace the sources of new viruses that spread widely among human beings, wild animals are often considered first, and the animals in domestication are rarely considered. As we know, the hunting of animals by human beings has always existed along with the progress of human civilization. It is difficult to explain the sudden emergence of highly pathogenic or infectious viruses from the habitual hunting and game-eating behaviors of human beings. The large-scale captive breeding of wild animals and hybrid improvement of varieties may be an important link in the process of viral transmission and evolution. This type of species variety, which differs from those found under natural conditions, provides an important premise for the variation and evolution of the virus. It also provides a convenient channel for spreading the virus to human beings and its rapid evolution because of its extensive contact with humans.

We have made many efforts in searching for the source of the virus, which spreads widely in human beings. However, we still do not know the source and the intermediate route of the sudden and large-scale virus outbreak. The important reason is that we may neglect the critical group of semi-wild animals domesticated by human beings, which may be an important direction for finding the intermediate host of the virus.
